# Bridging the diagnostic gap in diabetes 

**DOI:** 10.2471/BLT.25.293828

**Published:** 2025-11-08

**Authors:** Bianca Hemmingsen, Jennifer Manne-Goehler, Adriana Velazquez Berumen

**Affiliations:** a Department of Noncommunicable Diseases, Rehabilitation and Disability, World Health Organization, Avenue Appia 20, 1211 Geneva 27, Switzerland.; b Brigham and Women’s Hospital, Harvard Medical School, Boston, United States of America.; c Health Products Policy and Standards Department, World Health Organization, Geneva, Switzerland.

In 2022, an estimated 828 million people aged 18 years and older, about 14% of the global adult population, were living with diabetes, raising from a prevalence of 7% in 1990.^1^ This alarming figure underscores the growing burden of diabetes on health systems worldwide. Notably, the largest increases in prevalence were observed in low- and middle-income countries, highlighting the considerable challenge for health systems with fewer resources. In 2022, an estimated 445 million adults aged 30 years or older with diabetes (representing about 59% of this population) were not receiving any glucose-lowering pharmacological treatment. Most of the people with untreated diabetes had not been diagnosed, with undiagnosed cases accounting for 84–97% of untreated diabetes.^1^ This diagnostic gap is particularly large in low- and middle-income countries, where access to health-care services and diagnostic tools is limited. 

This gap reflects three major health systems challenges: limited access to diagnostic technologies; limited familiarity of health workers with diagnostic devices and testing algorithms for diabetes; and low public awareness of diabetes. First, several studies have shown limited access to diagnostic technologies relevant for the optimal care of diabetes. For instance, a systematic review about the availability and affordability of essential diagnostics for diabetes (mainly in the World Health Organization (WHO) African Region) found that glucometers were only available in about half of the clinics included in the study, and Haemoglobin A1c (HbA1c) diagnostics were available in about one quarter of the clinics, with data collection undertaken in a mix of public and private facilities.^2^ Diabetes is a condition for which point-of-care diagnostics could speed the scale-up of care. Positively, the development of accurate, robust point-of-care diagnostic technologies has continued to evolve. For instance, a prospective, quantitative, accuracy study evaluated the performance of two leading point-of-care diagnostic systems for HbA1c in rural Nepal, compared with local standard laboratory-based HbA1c tests.^3^ The study found robust accuracy in these conditions but highlighted ongoing usability challenges such as the need for substantial training on how to use it; a similar study recently completed in Indonesia demonstrated comparable findings.^4^ Hence, further testing and refinement of point-of-care diagnostic tests are needed, including implementation studies across a wider range of settings.

The second driver of the diagnostic gap is the limited training of key health workers in the principles of diabetes diagnosis and its complications. Prior research has shown the relatively weak understanding of diabetes diagnosis and management in certain low- and middle-income settings.^5^^–^^7^ For instance, a study of diabetes knowledge among health workers in Indonesia showed low scores in the range of 29–37% on diabetes clinical vignettes.^7^ A similar study in Islamic Republic of Iran revealed a lack of familiarity with common diagnostic thresholds for diabetes, including those based on HbA1c.^5^

The third challenge involves the low public awareness of diabetes, which has been well documented across multiple contexts. For example, in a survey of over 12 100 adults older than 30 years in Bangladesh, only one third were able to correctly identify a cause of diabetes and only approximately 15% had ever been tested.^8^ Similar findings regarding awareness of diabetes have been observed in other settings: for instance, in a survey in Poland, only about 17% (182/1051) of those surveyed reported a good level of knowledge about diabetes.^6^ This awareness gap likely contributes to the low rates of diagnosis.

Key actions to bridge these gaps include implementing national health policies that address noncommunicable diseases; establishing national diabetes guidelines and protocols; ensuring the availability of a trained health workforce at all levels; and providing insurance benefit packages that cover diabetes diagnosis and treatment. Policy-makers must also ensure access to insulin and related treatments necessary for the effective management of people with diabetes. Health systems must ensure the availability and affordability of high-quality diagnostic devices, medicines and related consumables (such as syringes, lancets and test strips) that are quality-assured by a regulatory agency, and that these are reliably procured and supplied to the end user. In addition, health systems should include data monitoring systems and indicators to measure performance in diabetes diagnosis and control.

WHO has responded to the increase in diabetes prevalence in several ways. In April 2021, WHO launched the Global Diabetes Compact with the aim of reducing the risk of diabetes and ensuring that all people who are diagnosed with diabetes have access to equitable, comprehensive, affordable and quality treatment and care. In May 2021, WHO Member States endorsed resolution WHA74.4 to strengthen the prevention and control of diabetes.^9^ One year later, Member States endorsed the first-ever global diabetes coverage targets. The targets include that by 2030, 80% of people with diabetes are diagnosed; 80% of those diagnosed achieve good glycaemic and blood pressure control; 60% of those with diagnosed diabetes who are older than 40 years receive a statin; and all people with Type 1 diabetes have universal access to affordable insulin and blood glucose self-monitoring.^10^ A month later, WHO published the *WHO list of priority medical devices for management of cardiovascular diseases and diabetes*,^11^ indicating that diagnostic devices are needed for diagnosis and monitoring at different levels of care, and therefore aligning with resolution WHA74.4 and the global coverage targets.

The *WHO package of essential noncommunicable disease (PEN) interventions for primary health care* recommends testing adults who present with symptoms of diabetes or are older than 40 years and overweight or obese for diabetes. Health systems and policy-makers must strengthen diagnostic capacities for timely and accurate identification of those who meet these criteria, increase access to treatment, and implement effective public health strategies to manage and prevent diabetes.

Next, in May 2023, the World Health Assembly endorsed resolution WHA76.5, *Strengthening diagnostics capacity*,^12^ which emphasizes the importance of diagnostic capacity, improving access and implementing targeted measures. An important strategy to implement the resolution is the promotion of the WHO *Model list of essential in vitro diagnostics*, which includes glucose and HbA1c tests, both essential for diagnosing and monitoring diabetes. Other priority activities under WHA76.5 include developing national diagnostics strategies, creating national essential diagnostics lists and expanding the scope of packages of essential diagnostic services. These activities should be prioritized by policy-makers and health authorities. This resolution also highlights the importance of point-of-care tests at the primary health-care level as well as at the community level, including self-testing, to increase access to and the affordability and use of diagnostics.^12^ The resolution also urges Member States, considering their national context, to invest in skilled workforce and in diagnostic services.

To support the procurement of quality diagnostics, WHO has developed technical specifications for the procurement of essential diabetes diagnostic devices such as glucose meters and HbA1c testing systems, which are publicly available in WHO’s medical devices information system, Medevis.^13^ The WHO prequalification programme is another initiative intended to close the diagnostic gap by listing quality-assured glucometers and HbA1c point-of care-testing. However, these diagnostic devices are not covered by many health insurance packages and the cost of the consumables, especially test strips, is a major challenge in low- and middle-income countries.^14^

Finally, as a first integral component of a comprehensive treatment and care system, enhanced diagnostic capacity for diabetes should be monitored through core health systems data. The WHO STEPwise Approach to Surveillance surveys offer one potentially important data source for assessing the diagnostic gap in diabetes, although the surveys conducted to date have been limited by geographic coverage and frequency of data collection. The global monitoring framework for diabetes prevention and control offers a comprehensive approach for countries to track diabetes prevention, care and outcomes in the domains of health system determinants, service delivery, risk factors and outcomes, including identifying opportunities to integrate data collection and digital technologies to improve data quality and accessibility.

Addressing the gaps in diagnosis and downstream treatment is essential for improving diabetes care and achieving these global diabetes coverage targets. The global gap in diabetes diagnosis remains a critical barrier to effective care, as undiagnosed individuals cannot access treatment and will be at high risk of complications, leading to additional burden on health systems and patients. Strengthening diagnostic capacity and related health system structures is key to scaling up diabetes care and improving lives worldwide. However, given current trends, the global coverage targets for 2030 are unlikely to be achieved without fast and coordinated action. Without such efforts, the diagnostic gap will persist, threatening progress and leaving millions without access to essential care.
